# A Real-Time, Non-Contact Method for In-Line Inspection of Oil and Gas Pipelines Using Optical Sensor Array

**DOI:** 10.3390/s19163615

**Published:** 2019-08-20

**Authors:** Santhakumar Sampath, Bishakh Bhattacharya, Pouria Aryan, Hoon Sohn

**Affiliations:** 1 Department of Civil & Environmental Engineering, KAIST, 291 Daehakro, Yuseong-gu, Daejeon 34141, Korea; 2 Department of Mechanical Engineering, Indian Institute of Technology Kanpur, Kanpur 208016, India

**Keywords:** in-line inspection (ILI) robot, non-destructive evaluation (NDE), real-time monitoring, optical sensor array, corrosion defects, pipeline network, smart PIG

## Abstract

Corrosion is considered as one of the most predominant causes of pipeline failures in the oil and gas industry and normally cannot be easily detected at the inner surface of pipelines without service disruption. The real-time inspection of oil and gas pipelines is extremely vital to mitigate accidents and maintenance cost as well as to improve the oil and gas transport efficiency. In this paper, a new, non-contact optical sensor array method for real-time inspection and non-destructive evaluation (NDE) of pipelines is presented. The proposed optical method consists of light emitting diodes (LEDs) and light dependent resistors (LDRs) to send light and receive reflected light from the inner surface of pipelines. The uniqueness of the proposed method lies in its accurate detection as well as its localization of corrosion defects, based on the utilization of optical sensor array in the pipeline, and also the flexibility with which this system can be adopted for pipelines with different services, sizes, and materials, as well as the method’s economic viability. Experimental studies are conducted considering corrosion defects with different features and dimensions to confirm the robustness and accuracy of the method. The obtained data are processed with discrete wavelet transform (DWT) for noise cancelation and feature extraction. The estimated sizes of the corrosion defects for different physical parameters, such as inspection speed and lift-off distance, are investigated and, finally, some preliminary tests are conducted based on the implementation of the proposed method on an in-line developed smart pipeline inspection gauge (PIG) for in-line inspection (ILI) application, with resulting success.

## 1. Introduction

Pipelines are the common agents used for the transportation of fluids, such as oil and gas, from one place (source) to another (customer). The pipeline is one of the most energy-efficient, reliable and economical ways to transport fluids over long distances in the order of thousands of kilometers. The transportation of oil and gas through pipelines has been rapidly increasing in recent years; only in India, for example, more than 13,000 km of oil and gas pipelines have been installed [1]. It has been reported that, considering the current trend, by the end of 2020, India will install approximately 16,000 km of oil and gas pipelines, additionally [2].

However, the safe operation of pipelines can be easily threatened by different factors such as time-dependent degradations (corrosion, oxidation, creep, etc.), construction defects, or static or dynamic loads originating from outside and inside of the pipelines and related to environmental issues (severe temperature conditions) [3,4,5,6]. More significantly, during the transportation of oil and gas, impurities such as carbon dioxide or water can cause corrosion on the inner wall of the pipelines. Corrosion can lead to a decrease in the effective wall thickness, which may ultimately result in rupture or leakage [7,8,9,10,11,12]. Corrosion defects are the most predominant cause of such pipeline failures, which may exist in various forms such as deposit corrosion, cavitation corrosion, uniform corrosion, pitting corrosion, etc. Corrosion accounts for about one quarter to two thirds of the total downtime in industries related to pipelines [9]. The failure of such pipelines under working conditions can result in human casualties, environmental damage and production losses. The failure of such factors in oil and gas pipelines have been reported in India [13,14] and other parts of the world [15,16,17]. However, the preventive maintenance and inspection of the hazardous oil and gas pipelines are quite challenging since most of these pipelines are buried underground. Also, external real-time inspection of long pipelines (e.g., 13,000 km) is nearly infeasible. Therefore, inspection robot-based internal health is necessary to guarantee the safe operation of pipelines.

In the last few decades, pipeline inspection gauges (PIGs) have become more prevalent for in-line inspection (ILI) and non-destructive evaluation of the pipelines [10,18,19]. The advanced versions of these autonomous systems, also called “smart PIGs”, can move inside pipelines and measure irregularities that may represent corrosion, cracks, joints, deformation (e.g., dents, pipe ovality), laminations or other defects (e.g., weld defects) in the pipeline. The most common ILI methods that have been installed on smart PIGs and confirmed to be successful for pipeline inspection are magnetic flux leakages (MFL) [20], ultrasonic transducers (UT) [21], electromagnetic acoustic transducers (EMAT) [22] and eddy currents (EC) [23]. However, certain constraints seriously limit the practicality of the aforementioned methods. In the MFL method for instance, it is very difficult to effectively saturate the entire cross-section of the pipeline with magnetic flux, and also the servicing process involves frequent calibration and complete analysis. Moreover, the method is not suitable to inspect non-ferrous pipelines [24,25,26]. The UT method works well in liquid pipelines; however, the application in gas pipelines is not common since it requires liquid coupling between the transducer and the surface of the pipeline [27]. UT is more suitable for thick-wall pipelines rather than thin-wall pipelines (less than 7 mm) [28]. Echo loss is another major challenge reported in the literature [29]. The EMAT method cannot be used in non-conductive materials such as plastics or ceramics, and it is not suitable for long pipeline inspection, which requires high power and complex signal processing in real-time [30]. This method faces challenges for high-speed scanning in pipelines, and it can be applicable up to 2.5 m/s [31]. The EC method requires deep magnetic penetration in ferrous pipelines, and the major drawback is the spacing problem, which occurs while mounting the sensor array on the circumference of the smart pig [32,33]. In addition, recently, a few ILI methods have also been developed for the inspection of pipelines such as closed-circuit television (CCTV) [34] and mechanical contact probe (MCP) [35]. In the case of the CCTV method, the high power supply and lack of visibility inside the long pipelines are the drawbacks [36]. The MCP method can inspect only convex defects such as deposit corrosion. It is not suitable for cavity corrosion or metal loss corrosion, and the friction involved in the inspection process is a major risk [35].

This paper presents a new, real-time, non-contact method for the inspection of internal corrosion defects in gas pipelines using an optical sensor array. The method utilizes an optical sensor array consisting of emitter elements, light emitting diodes (LEDs), and receiver elements, light dependent resistors (LDRs). The output signal is further processed with a discrete wavelet transform (DWT) for noise cancelation and feature extractions. The estimated lengths and heights of defects for different physical parameters are investigated. The parameters include inspection speed and lift-off (referred to as the distance between the pipeline surface and the sensor surface). The preliminary experiments are conducted successfully based on the installation of the proposed method on an in-house developed smart pig. The proposed method has significant capabilities and the potential to overcome the aforementioned limitations of the conventional and current ILI methods. The significance of the developed system lies in being a non-contact, real-time, potential method for ILI application.

The paper is organized as follows. Section 2 introduces the proposed method and provides a theoretical background. Experimental studies, including the designed experimental setup, the specimens as well as the signal processing for noise cancelation, are described in Section 3. Section 4 presents the performance of the proposed system regarding the detection of corrosion-type defects as well as the preliminary test outcome for ILI application. The conclusions and summary of outcomes of the current research along with the future directions are presented in Section 5.

## 2. The Method

**Figure 1:**
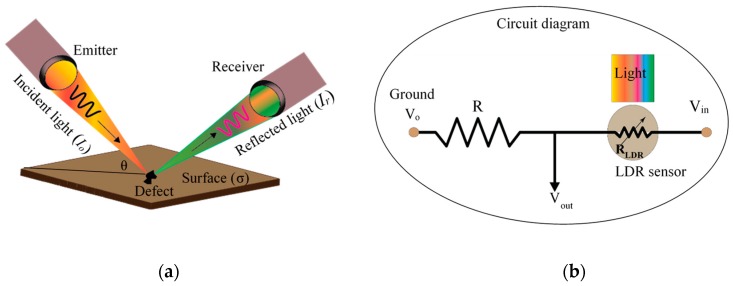
Complete procedure of the proposed method: (**a**) schematic picture of the optical sensor working principle and (**b**) the built circuit diagram.

The proposed method is based on the absorption of emitted (reflected) light and the utilization of optical sensor array. Light from the emitter travels through the medium (oil or gas) and is reflected from the inner surface of the pipeline, as shown in Figure 1a. The reflected light can be further analyzed to extract the information related to surface irregularities (defects).

### 2.1. Background and Working Principle

The relationship between the intensity of reflected light *I_r_* and the roughness of the sample surface (*σ*) can be described in the following equation [37]:

(1) $$\frac{I_{r}}{I_{o}}=kexp\left[-{\left(\frac{4\pi \sigma \cos\theta }{\lambda }\right)}^{2}\right]$$

where *k* represents the material constant, *θ* is the incident angle of light (which is normal to the surface), *λ* is the wavelength of the LED emitter (e.g., green light 510 nm) and Io stands for the intensity of the reflected light from a defect-free surface (smooth). Equation (1) shows how the defect affects the intensity of the reflected light. The defects change the surface roughness (*σ*) of the pipeline and, consequently, change the intensity of the reflected light [38]. The relationship between the intensity of light (*I*) and the resistance of the LDR sensor (*R_LDR_*) can be described by [39,40]:

(2) $$R_{LDR}=R_{\infty }-k_{1}\ln I$$

where $R_{\infty }$ is the resistance of the sensor, considering *I_r_,* and the respective resistances can be described by:

(3) $$R_{r}=R_{\infty }-k_{1}\ln I_{r}$$

(4) $$R_{o}=R_{\infty }-k_{1}\ln I_{o}$$

The subtraction of Equation (4) from Equation (3) results in:

(5) $$R_{r}=R_{o}-k_{1}\left(\frac{I_{r}}{I_{o}}\right)$$

Considering Equations (1) and (5), they can be written as:

(6) $$R_{r}=R_{o}-k_{1}\ln\left(kexp\left[-{\left(\frac{4\pi \sigma \cos\theta }{\lambda }\right)}^{2}\right]\right)$$

Equation (6) correlates the resistance of reflected light and the surface roughness of the pipeline. The circuit diagram of the sensor was built for this study as shown in Figure 1b. The sensor is connected to the input voltage (*V_in_*) through a fixed resistor in series. The resistance of the LDR sensor changes with the variation of the light intensity, which can be converted to output voltage (*V_out_*). This output voltage across the sensor is measured using the voltage divider rule by Equation (7), where *R* is the resistance of the fixed resistor.

(7) $$V_{out}=V_{in}\left(\frac{R_{LDR}}{R_{LDR}+R}\right)$$

### 2.2. Design of the Optical Sensor Array System

**Figure 2:**
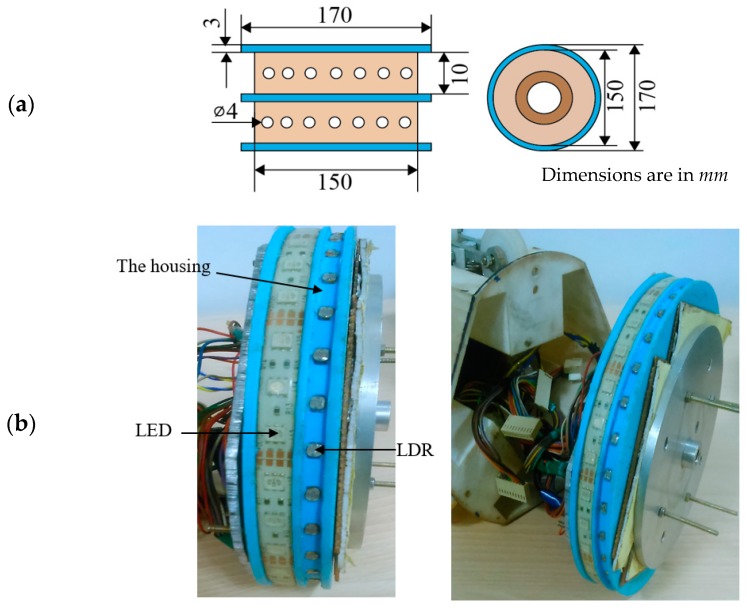
Schematic of the optical sensor array housing system; (**a**) side view and top view of the 2-D optical sensor array system, (**b**) photographs of a side view and the optical sensor array system.

The LED array emitters and LDR array sensors are mounted on a housing system with the configuration and dimensions shown in Figure 2. The diameter of the housing is 170 mm, and a 10-mm (diameter of the gas pipeline is 190 mm) gap is kept for the gas to flow. There are 28 emitters and 32 LDR sensors that are mounted over the circumference of the housing. The distances between two adjacent emitters and sensors are kept at ~6 mm and ~5 mm, respectively. The emitters are powered by a 12-V PSD (Digital Power Supply) DC power supply linear mode PS-305D device, and the sensors with a 5-V DC supply through a microcontroller (Arduino mega 2560). The relative response of the cadmium sulfide (CdS) LDR sensor has high sensitivity to the green light LED (broadband emission with wavelength at 510 nm) [41]. In this paper, the CdS LDR sensor and green light LED are used for sensing the surface defects. The LED emitters are of the EST-312-G20 model, made by Elstar Electronic Co., Ltd. in Guangdong, China, and the max. power consumption is 1.632 Watt for a 170-mm length of the strip. The LDR Receivers are of the GL55 Series, made by Nanyang Senba Optical And Electronic Co., Ltd., Shenzhen, China. The electrical properties of the optical sensor array are listed in Table 1. The proposed sensors and emitters are relatively inexpensive (less than 3$ (USD) for each pair of sensor and emitter) and require low power (approx. 2.5 mW for each sensor). 

## 3. Experimental Studies

### 3.1. Specimen Description

**Figure 3:**
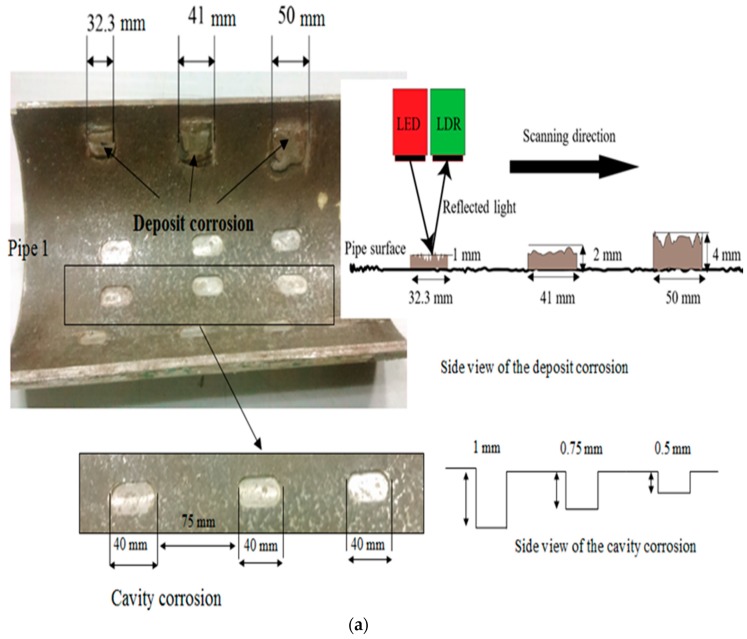

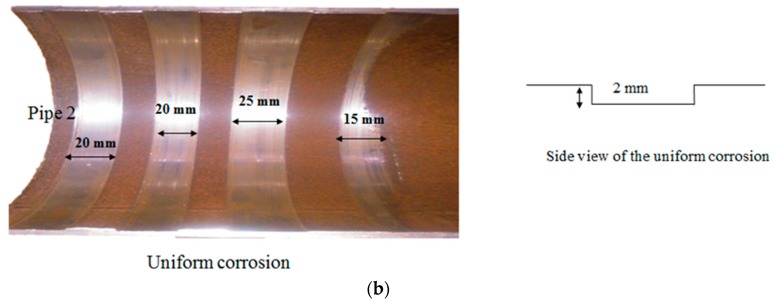
The specimens for the experimental studies with dimensions and three different types of corrosion. (**a**) deposit and cavity corrosions, (**b**) uniform corrosion

Two segmented steel pipelines with 190-mm inner diameter and 8-mm wall thickness are used as specimens for the experimental studies. The average chemical compositions of the steel pipeline specimens are given in Table 2. Three types of corrosion defects, namely, deposit corrosion, cavitation corrosion and uniform corrosion, are considered. These defects are artificially milled and deposited on the inner surface of the pipeline based on the specifications of the common defects reported in the literature [10,42,43,44]. Initially, the deposit corrosion defect is used for the calibration of the inspection method. Subsequently, the cavitation and the uniform corrosion defect are considered to confirm the effectiveness of the method is shown in Figure 3.

### 3.2. Experimental Setup and Procedure

**Figure 4:**
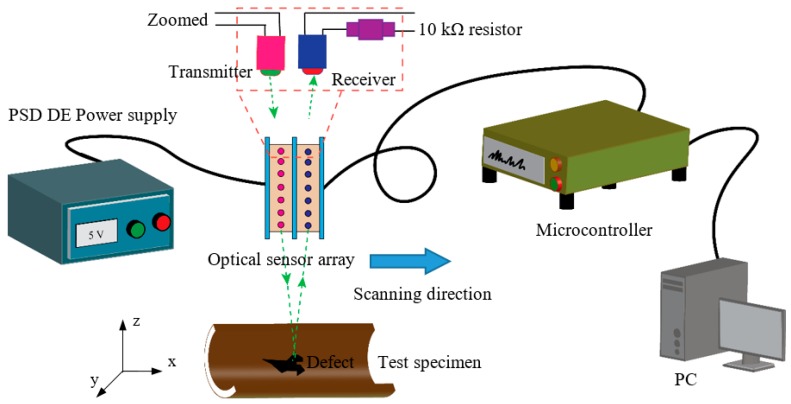
Schematic diagram of the self-designed experimental setup with interconnections.

The experiments are conducted in a self-designed laboratory testbed. The testbed consists of an optical sensor array housing system, rack and pinion mechanism to control the housing system, power supply and microcontroller. The designed optical sensor array can be controlled by a microcontroller and moved over the pipeline segments. A 12-V DC power supplier is utilized to supply the power to the LED emitters. The 10-kΩ resistor from one side is connected to the sensor in series, and from the other side to the microcontroller, to measure the voltage in the sensor. For the scanning of the segmented pipeline surfaces, the optical sensor array housing system moves linearly using a rack and pinion mechanism. A LabVIEW based program is developed to control the rack and pinion mechanism and also the microcontroller for the data acquisition process. The positioning of the sensor measures the intensity of the reflected light from an area that is separated by a specific distance, 5 mm from the emitters. To increase the reflected intensity of light, the angle of the incident emitter was set at zero, normal to the surface of the pipeline. Both emitters and receivers send and receive the light simultaneously. In other words, all are “Switched ON” at the same time. A schematic diagram of the experimental setup for the optical sensor array is shown in Figure 4.

**Figure 5:**
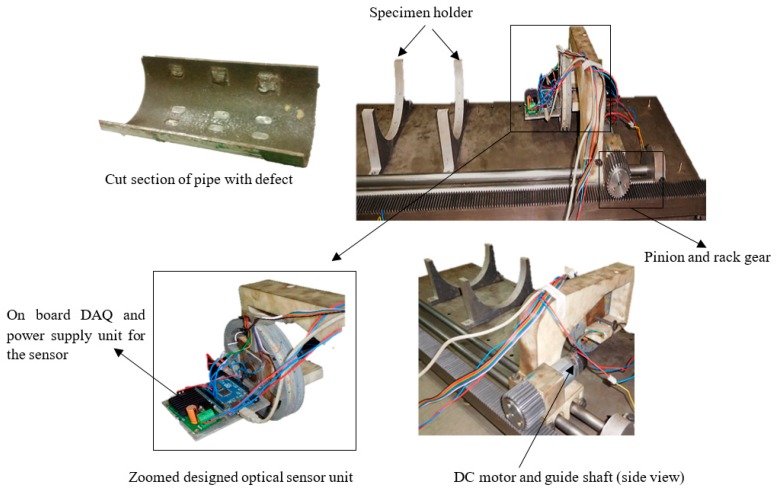
Picture of the self-designed laboratory testbed with different parts of the optical sensor array housing system, rack and pinion mechanism and the segmented pipeline.

Figure 5 shows the rack and pinion mechanism for the inspection of pipeline segments.

Here, the lift-off is referred to as the distance between the surface of the sensor and the surface of the pipeline, which is kept constant throughout the experiment. LabVIEW software is used to control the angular speed and direction of the DC motor, which is connected to the pinion. Initially, the DC motor rotates at 35 rpm, and the corresponding inspection speed of the optical sensor array system is 2.9 mm/s. Data are acquired for this inspection speed for different lift-off cases: case 1 is 20-mm lift-off and case 2 is 30-mm lift-off. Similarly, the experiments are performed and data recorded at different inspection speeds: 7.3, 11.0 and 13.0 mm/s for 15- and 20-mm lift-off cases, respectively. Experiments are carried out in the dark-room laboratory and during the experiments, all lights are turned off to simulate the real pipeline condition and minimize unnecessary errors from ambient lights.

### 3.3. Discrete Wavelet Transforms for Sensor Signal De-Noising

The common techniques for de-noising of recorded signals (e.g., signal average, median filtering and adaptive filtering) fall short in the case of non-stationary signals [45,46]. To improve the signal to noise ratio (SNR) for non-stationary signals, techniques such as discrete wavelet transform (DWT) [47], empirical mode decompositions [48] and Savitzky–Golay filtering [49] have been reported to be successful. Amongst these techniques, DWT is a versatile signal-processing tool for many engineering and scientific applications and suitable for both non-stationary and stationary signals [50]. The DWT method decomposes a sensor signal into high-frequency and low-frequency components using filter flanks. The high-frequency component is called the detail coefficient, and the low frequency is referred to as the approximation coefficient. The DWT of a signal *x(t*) is given [51] by the following equation:

(8) $$X_{DWT}=\overset{\infty }{\underset{-\infty }{\int }}x\left(t\right)2^{m/2}\psi \left(2^{m}t-k\right)dt$$

where *x*(*t*) is the sensor time-domain signal, and *m* and *k* are the wavelet function. The acquired sensor signal is passed through a low-pass filter and a high-pass filter at level one, and downsampled by a factor of two to obtain the approximation coefficients and detail coefficients [52]. In the next level, the low-pass signal obtained from level one is passed through a similar filter again, and sub-down-sampled by a factor of two to obtain the approximation coefficients and detail coefficients. This procedure is repeated for subsequent levels, the output of the previous level feeding onto the successive level. This step-by-step procedure is known as multiresolution analysis (MRA), and a detailed review is provided in [53]. Wavelet decomposition is carried out until the optimum level of decomposition is achieved by the given input sensor signal. A sensor signal estimation technique called wavelet thresholding has signal de-noising capabilities, and wavelet shrinkage operation is categorized into two thresholding de-noising methods, hard and soft wavelets. The soft-wavelet threshold de-noising method makes a continuous distribution of the remaining coefficients centered on zero by scaling them. The equation for the soft-wavelet threshold de-noising method [53,54] is given as follows:

(9) $$\hat{X}=\{\begin{array}{c} y\left(t\right)=sign\left(x\left(t\right)\right)\left|x\left(t\right)\right|-T,\;if\;\left|x\right|\geq T\\ 0,\;if\;\left|x\right|<T \end{array}$$

The hard-wavelet threshold de-noising method, as in [54], is given by:

(10) $$\hat{X}=\{\begin{array}{c} y\left(t\right)=x\left(t\right),\;if\left|x\right|\geq T\\ 0,\;if\;\left|x\right|<T \end{array}$$

where *x*(*t*) is the original signal, *y*(*t*) is the signal after threshold and *T* is the threshold value. Selections of threshold value play a significant role in processing sensor signals. There are different types of thresholding rules used to calculate the threshold value for de-noising applications. Global or universal thresholding [54], minimax [55] and hybrid threshold rules are used in the present work for de-noising the sensor signal. The global threshold is [56] given by:

(11) $$T=m\sqrt{2\log\left(N\right)}$$

(12) $$m=\frac{MAD}{0.6745}$$

where *m* is the noise intensity of the signal, MAD is the median of the wavelet coefficient and *N* is the total number of samples in the analyzed sensor signal. The inverse wavelet transform is used for the reconstruction of the sensor signal by passing it through a series of low-pass filters and high-pass filters. The reconstruction of the sensor signal is performed from DWT coefficients and the inverse discrete wavelet transform (IDWT), as in [51], is given by:

(13) $$X_{IDWT}\left(t\right)=\overset{\infty }{\underset{-\infty }{\int }}\overset{m=\infty }{\underset{m=-\infty }{\sum }}\overset{k=\infty }{\underset{k=-\infty }{\sum }}x_{k}^{m}2^{m/2}\psi \left(t-k\right)dt$$

### 3.4. Evaluation of Sensor Signal De-Noising

The de-noising of the sensor signal by DWT may not continuously remove noise content in the sensor signal since beyond a particular level of decomposition, important information may also be removed. There are few quantitative parameters that can be used to evaluate the quality of the reconstructed sensor signal. In this case, SNR and root mean square error (RMSE) parameters are adopted to determine the optimum level of decomposition, and the expressions are given by [57,58]. SNR and RMSE provide measures for the strength of the sensor signal [59] and can be calculated using the following equations:

(14) $$\mathrm{SNR}=20\log_{10}\left(\frac{\sum_{t=1}^{N}{\left(x\left(t\right)\right)}^{2}}{\sum_{t=1}^{N}{\left(x\left(t\right)-\hat{x}\left(t\right)\right)}^{2}}\right)$$

(15) $$\mathrm{RMSE}=\sqrt{\frac{1}{N}\overset{N}{\underset{t=1}{\sum }}{\left(x\left(t\right)-\hat{x}\left(t\right)\right)}^{2}}$$

where *x*(*t*) is the original noisy sensor signal and $\hat{x}$(*t*) is the de-noised sensor signal. *N* is the total number of samples in the sensor signal. In Section 4, the experimental results are presented based on the DWT.

## 4. Results and Discussion

### 4.1. Optimization of the Parameters and Damage Detection

**Figure 6:**
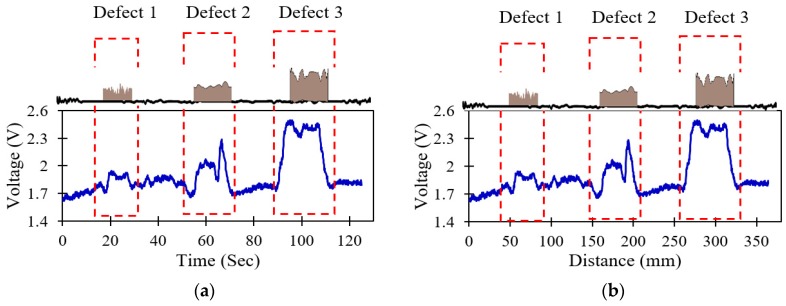
The records acquired sensor signals in the presence of deposit corrosion for 20-mm lift-off and 2.9 mm/s; (**a**) original time-domain sensor signal, (**b**) corresponding distance sensor signal.

The effect of SNR and RMSE by different types of wavelet packets, as well as the effects of the different levels of decompositions, threshold de-noising methods and thresholding rules on SNR and RMSE, are investigated in this section. Initially, the deposit corrosion defect with three different dimensions is considered to calibrate the physical parameters. The recorded sensor signals in the time domain at 2.9-mm/s inspection speed with a lift-off distance of 20 mm are shown in Figure 6. The intensity of reflected light is found to be prominent and constant when there are no defects on the surface of the pipeline. On the other hand, when there are deposit corrosion defects, then the intensity of reflected light is substantially reduced due to increased scattering.

**Figure 7:**
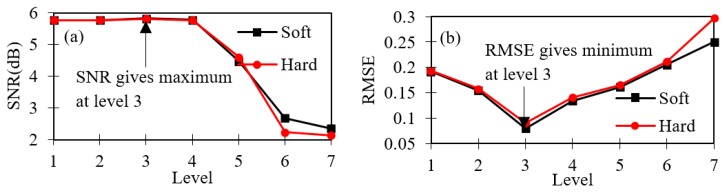
At each level of decompositions: (**a**) signal to noise ratio (SNR) values, (**b**) root mean square error (RMSE) values.

Different levels of decompositions are carried out using different threshold de-noising methods and thresholding rules, as shown in Figure 7. Table 3 shows the computed values of SNR and RMSE for different levels of decomposition, soft and hard de-noising methods with different threshold rules. Effective information of the sensor signal retained is considered to be at the highest value of SNR and the lowest value of RMSE, accordingly.

The maximum value of SNR (5.82144) and the minimum value of RMSE (0.08) are reached at decomposition level three. The corresponding mother wavelet db10 packet is used. It is observed that at decomposition level three, the soft de-noising method and universal threshold rule are the optimum choices. Followed by determining the optimum wavelet decomposition level, with the soft de-noising method and universal threshold rule, the selection of the wavelet packet is carried out. The appropriate selection of the mother wavelet is directly related to the effect of de-noising (results). The Daubechies (db), Harr, Biorthogonal (bior), Symlets (Sym) and Coiflets (Coif) mother wavelets have been used along with a soft-threshold de-noising method and universal threshold rule to decompose the sensor signal. The SNR and RMSE values are calculated for different types of mother wavelets at the level of three decompositions, as shown in Table 4.

**Figure 8:**
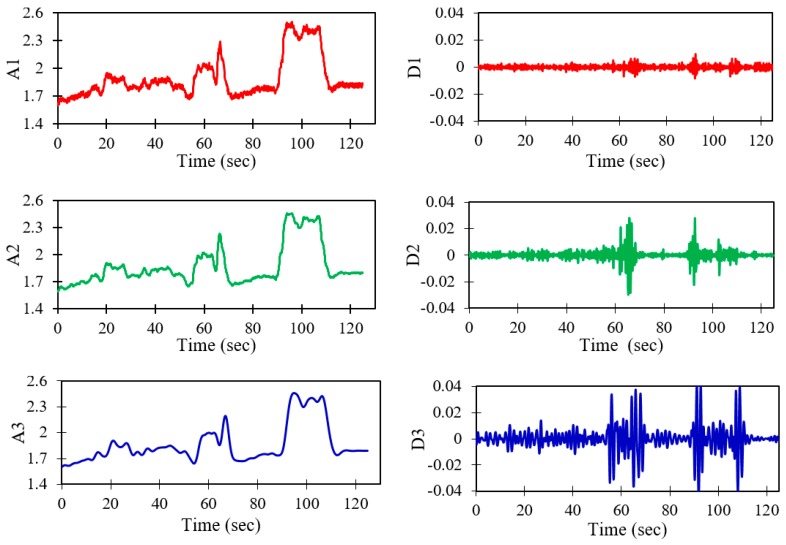
Approximation and detailed coefficients of the sensor signal at 2.9 mm/s.

**Figure 9:**
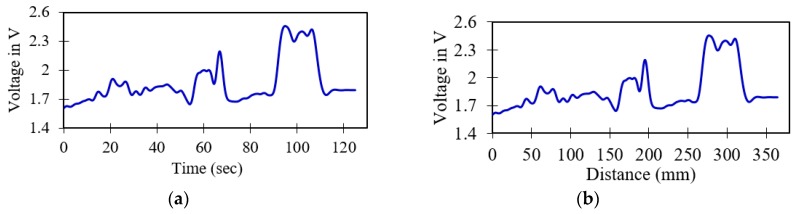
De-noised acquired sensor signal for deposit corrosion defects scanned at 2.9 mm/s for 20-mm lift-off; (**a**) time-domain sensor signal, (**b**) corresponding distance signal.

The SNR has reaches a maximum of 5.82144, and the corresponding minimum of the RMSE is 0.08, obtained for wavelet db10 with the application of the soft de-noising method and universal threshold rule at the third level of decomposition. The result of the DWT is a multilevel decomposition, where the sensor signal is decomposed into approximation coefficients and detail coefficients at each level. Figure 8 shows the three different levels of approximate coefficients (identified by A1–A3) and detailed coefficients (identified by D1–D3). This procedure is continued for each level of wavelet de-noising until the maximum SNR and minimum RMSE are achieved, as shown in Figure 9.

**Figure 10:**
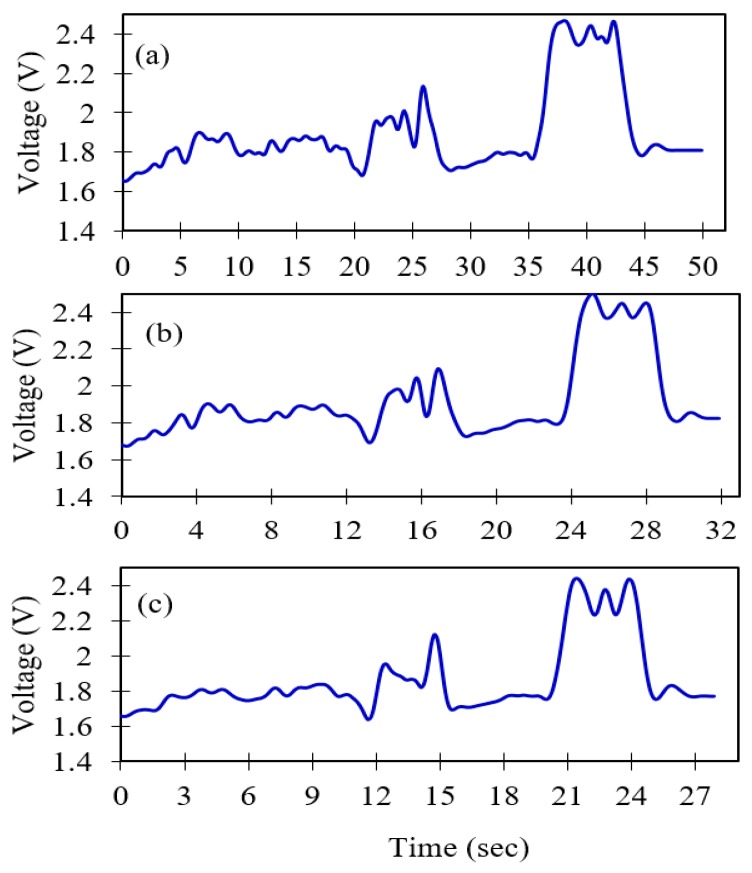
Discrete wavelet transform (DWT) de-noised sensor signals at different inspection speeds at the third decomposing level for 20-mm lift-off; (**a**) 7.3 mm/s, (**b**) 11 mm/s and, (**c**) 13 mm/s.

Similarly, sensor signals are de-noised at level three of DWT using the soft -threshold de-noising method and universal threshold rule for different inspection speeds of the optical sensor array system, at 7.3 mm/s, 11 mm/s and 13 mm/s, respectively, as shown in Figure 10a–c.

**Figure 11:**
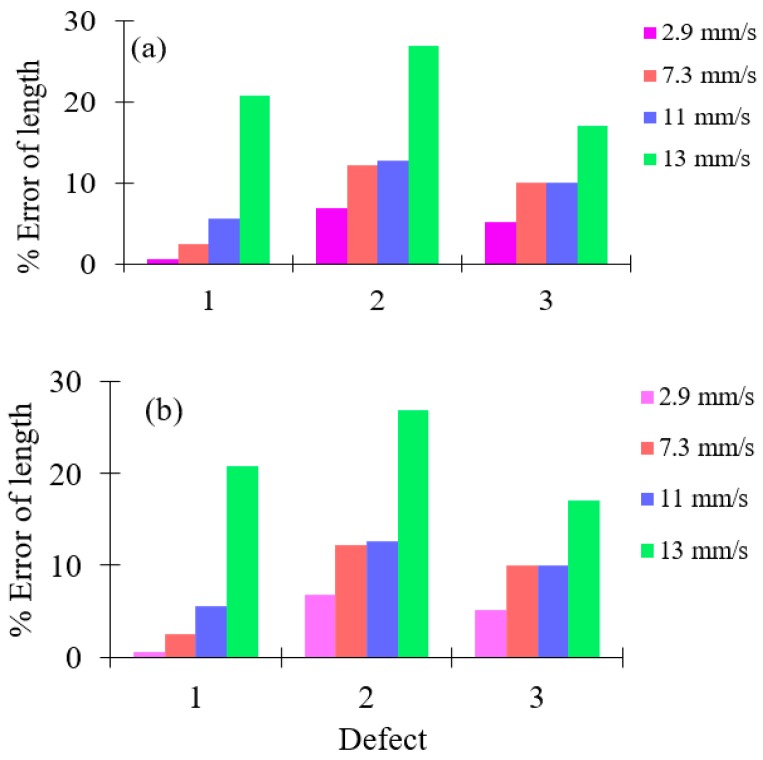
Percentage of error length at different inspection speeds for the different lift-offs; (**a**) 20-mm lift-off and, (**b**) 30-mm lift-off.

The length of deposit corrosion defects is measured at different inspection speeds for different lift-offs, and the percentages of error between actual and experimental defect lengths are estimated and presented in Table 5. The highest lift-off may affect the accuracy of the inspection of the optical sensor array system. Therefore, lift-off between the optical sensor and the pipe surface must be kept as close as possible to increase the accuracy of the inspection. From Figure 11, it is evident that when the inspection speed of the optical sensor array system is increased, the percentage of error between the actual and experimental estimation of defects is also increased.

**Figure 12:**
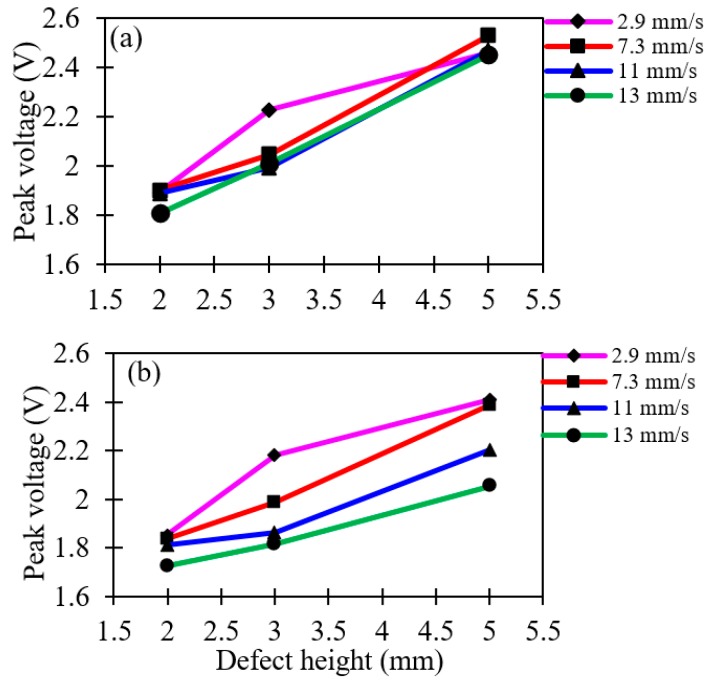
Peak voltage corresponding to the height of deposit corrosion defects for different lift-offs at different inspection speeds; (**a**) 20 mm and, (**b**) 30 mm.

The peak voltages recorded for different heights of defects are presented in Figure 12. It is observed that the maximum peak voltage is recorded for a 4-mm height of deposit corrosion defect. This is due to more reflected light falling on the sensor when the heights of defects are at the maximum. Finally, it is observed that the optimum physical parameters correspond to an inspection speed of 2.9 mm/s and a 20-mm lift-off distance, which gives the minimum percentage of error and the highest peak voltage.

### 4.2. Cavity Corrosion Detection

**Figure 13:**
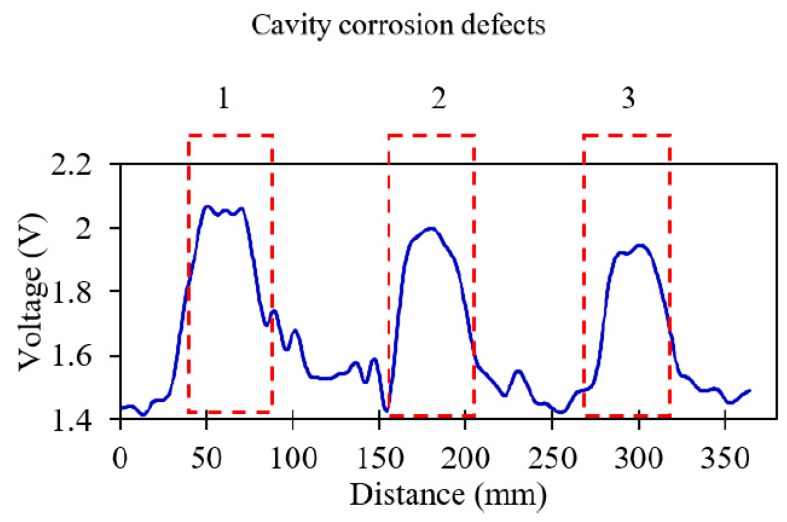
De-noised distance sensor signal for cavity corrosion defects scanned at 2.9 mm/s and 20-mm lift-off.

A similar analysis, using the previously optimized parameters, was conducted for the same specimen but detecting cavity corrosion on the surface of the same segmented pipeline. Three cavity corrosions with different depths (1, 0.75 and 0.5 mm) were milled to investigate the sensitivity of the proposed method to depth variation. Sensor signals de-noised at level three for a soft-threshold de-noising method with the mother wavelet of db10, universal threshold rule at 2.9 mm/s and 20-mm lift-off are shown in Figure 13. The location of the cavity corrosions and the corresponding sizes are marked on the figure. Similar patterns are observed for the cavity corrosion defects at different locations. The disturbance in the output voltage may be due to a slight vibration of the testbed. The associated errors and the peak voltage values of the obtained signals from the sensor are presented in Table 6.

The sensitivity of the method to depth variation can be clearly seen in the peak voltage values of the cavity corrosions. In other words, the intensity of the defect has a direct effect on the peak voltage; hence, the peak value in this case corresponds to the cavity corrosion with the greatest depth. The errors for the length of the defects are less than 5% in the case of cavity corrosions.

### 4.3. Uniform Corrosion Detection

**Figure 14:**
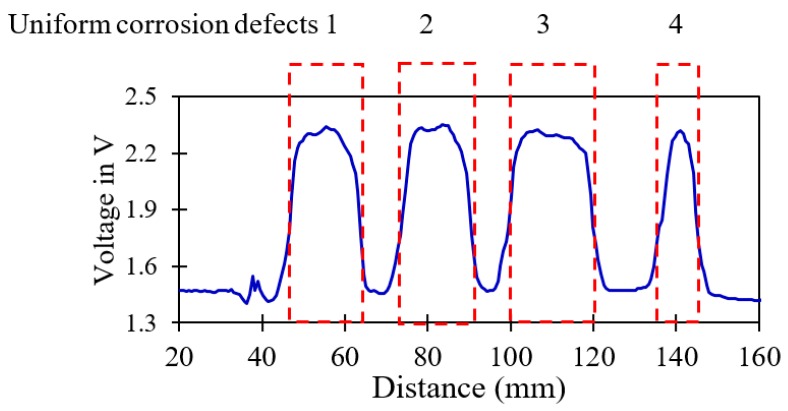
De-noised distance sensor signal for uniform corrosion defects with various widths at 2.9 mm/s and 20-mm lift-off.

Four uniform corrosion cases with different widths (20, 20, 25 and 15 mm) are created on the inner surface of the second segmented pipeline to observe the operation of the proposed method in the presence of width variation. Sensor signals de-noised at level three for a soft-threshold de-noising method with a mother wavelet of db10, universal threshold rule at 2.9 mm/s and 20-mm lift-off are shown in Figure 14. The locations of the cavity corrosions and the corresponding sizes are marked on the figure. The same as the previous case studies, similar patterns are observed for the uniform corrosion at different locations. The associated errors and the peak voltage values of the recorded signals from the sensor are presented in Table 7.

Due to the fact that the depths of the uniform corrosions are the same (2 mm), the peak voltage values are exactly the same. However, in terms of the width variation, the method is confirmed to be sensitive to detect width variation as small as 5 mm. The associated errors regarding the length of the defects are less than 10% in these cases.

### 4.4. Feasibility Test for Real-World Application

**Figure 15:**
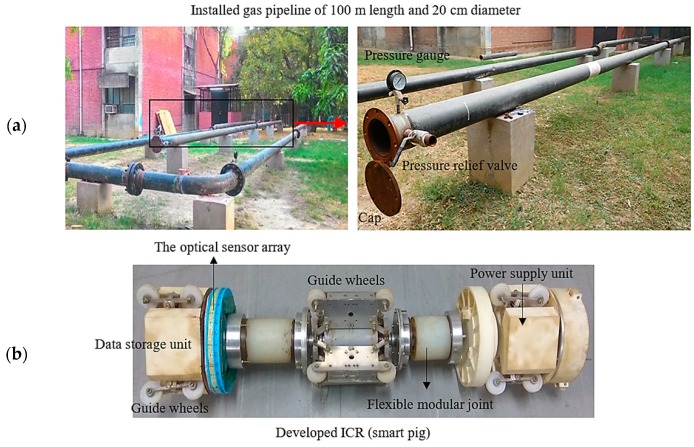
Images of the field test for in-line application; (**a**) gas transporting pipeline, (**b**) the designed PIG.

To observe the performance of the proposed method for in-line application, a field test was conducted for a real pipeline network developed at the institute. The proposed method is implemented on an in-house developed in-line crawler robot (ICR), also called PIG. The PIG is used for inspection purposes, and it is propelled by flow with the speed range up to 5 m/s. Since the robot flows along with the fluid, there is no hindrance to fluid flow. The PIG is equipped with an optical sensor array, odometers, microcontroller (Arduino Mega 2560), LabVIEW interface and a battery for power supply to the entire system. The steel pipeline with an inner diameter of 190 mm and a length of 100 m, with working gas of compressed air, was selected. The pipeline has been reported to have no defects. The complete test setup and the developed ICR are shown in Figure 15. The working pressure inside the pipeline for the test is 2 bar, and the duration of the inspection test is approximately 2.27 h with the speed of the PIG set to 10 mm/s.

**Figure 16:**
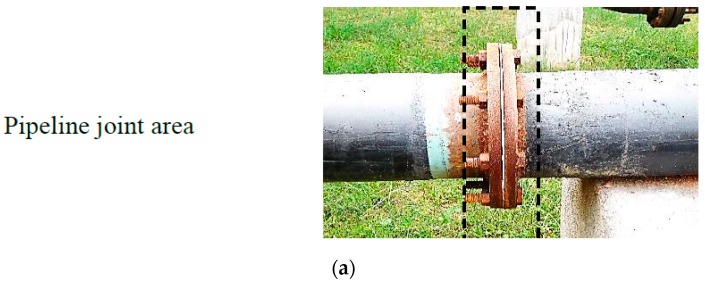

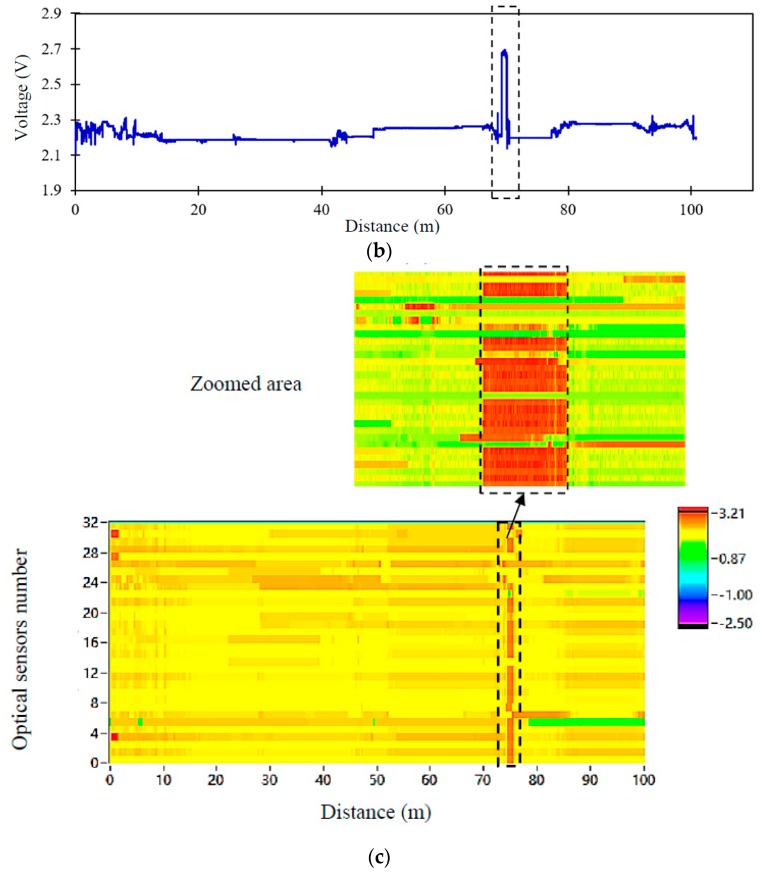
The results for the real-world application using the proposed method; (**a**) photo of the pipeline joint, (**b**) output voltage of the sensor for the whole pipe inspection, (**c**) 2D image of the scanned pipeline with zoomed abnormality area at a location between 70 m and 80 m.

The obtained results are presented in Figure 16. This plot corresponds to the signal recorded by one of the optical sensor arrays. In total, 32 optical sensor array signals are mapped into two dimensional (2D) images. In the mapping image, the y-axis is the number of optical sensor arrays vs. pipeline distance in the x-axis. The mapped 2D image can be utilized for quick visual inspection and extracting the information on the entire circumference of the pipeline as well as the length. The image from the optical sensor array shows drastic change at the location of the joints.

The output voltage from the sensor shows high amplitude fluctuations as well as jumps in various locations, which may be due to the significant vibration of the PIG. This problem should be studied further, and the optimized parameters such as lift-off distance, speed and proper level of de-noising have to be adjusted in the in-line inspection application. The main outcomes of the current research, which highlight the significance, current limitations and recommendations, are summarized in the conclusions.

## 5. Conclusions

A new, non-contact optical sensor array method for real-time inspection of pipelines is presented. Experimental studies are conducted considering corrosion defects with different features (three types; deposit, cavity and uniform) and dimensions to confirm the effectiveness and accuracy of the proposed method. The optimum parameters are experimentally and analytically selected to achieve maximum SNR and minimum RMSE. From the experimental results, the sizes, as well as the locations of the corrosion defects, were detected with less than 10% error. The proposed method showed effectiveness and sensitivity to the presence of defects and variations in dimensions. The method is cost-effective, non-contact and allows versatile inspection, and it can be integrated and installed on an ILI PIG. The proposed method has potential capabilities and flexibility for real-world application and in-line inspection purposes, regardless of the pipe material, dimensions or service. The current research is partially motivated by the growing expectations from pipeline industries and presented a candidate method for the in-line and real-time inspection of pipelines. For the next step, the performance of the method needs to be observed for several situations such as a pipeline network with multiple confirmed corrosion defects during service, significant temperature variations (outside the range of the sensor operation) and considering oil instead of gas (the effect of viscosity). Also, further studies are warranted for real-time, in-line inspection applications in terms of the data acquisition rate, storage and advanced efficient wireless data transfer without time delay. The PIG is capable of being equipped with a wireless robot tracking system such as X-bee (pro-S1 PCB antenna model), by which the position of the robot and locations of the probable defects can be obtained in real-time. Two X-bee modules are used to communicate with each other, where one is for the transmission and the other functions as the receiver. The transmitter X-bee is mounted on the microcontroller board, and the data are received by the receiver X-bee via RF (radio frequency) waves using the ZigBee protocol. The receiver X-bee is connected with another microcontroller and the data are sent to a PC using the LabVIEW interface.

## Figures and Tables

**Table 1:** Electrical properties of the designed optical sensor array.

Parameters	Light Emitting Diode (LED) Emitter	Light Dependent Resistor (LDR) Sensor
Dimension	4 × 8 × 2.4 mm	6.5 × 5.5 × 4 mm
Voltage (V)	12 V	5 V
Power Consumption	24 mW	-
Dark Resistance	-	1 MΩ
Power Dissipation	-	100 mW
Operating Temperature	−30 °C~85 °C	−30 °C~70 °C
Response Time	-	Rise time 20 ms, Decay time 30 ms
Relative Sensitivity of CdS for the Green LED	-	100%

**Table 2:** Chemical composition of the steel pipeline in mass%.

Chemical Content in Steel Pipe	P	Fe	Pb	C	Si	Ti	Cr	Mn	Co	Ni
mass%	0.03	92.26	0.128	6.088	0.18	0.032	0.066	0.974	0.152	0.086

**Table 3:** Effect of decomposition level and de-noising on the SNR and RMSE of the sensor signal.

Level of Decompositions	Method of De-Noising	Thresholding Rule					
		Hybrid		Universal		Minimax	
		SNR (dB)	RMSE	SNR (dB)	RMSE	SNR (dB)	RMSE
1	Soft	5.77094	0.197	5.77094	0.192	5.77092	0.191
	Hard	5.77087	0.192	5.77088	0.193	5.77084	0.192
2	Soft	5.77147	0.158	5.772	0.154	5.77175	0.153
	Hard	5.77093	0.154	5.77125	0.158	5.77102	0.152
3	Soft	5.81179	0.081	5.82144	0.08	5.80638	0.087
	Hard	5.80036	0.094	5.80471	0.091	5.77124	0.099
4	Soft	5.77375	0.135	5.77838	0.134	5.77667	0.135
	Hard	5.77101	0.140	5.77276	0.14	5.77204	0.146
5	Soft	5.30031	0.167	4.48544	0.161	4.69869	0.162
	Hard	5.29639	0.169	4.60401	0.165	4.65698	0.164
6	Soft	4.56004	0.204	2.67128	0.205	3.23969	0.205
	Hard	4.42163	0.212	2.2419	0.211	3.16649	0.216
7	Soft	2.35312	0.253	2.35758	0.25	3.23001	0.253
	Hard	2.35003	0.296	2.13721	0.297	3.14142	0.297

**Table 4:** The effects of de-noising by different mother wavelets.

Wavelets	SNR	RMSE	Wavelets	SNR	RMSE	Wavelets	SNR	RMSE
db2	5.44103	0.084	Haar	4.85419	0.083	bior5_5	5.74352	0.120
dp3	5.37424	0.092	bior1_3	5.38377	0.087	bior6_8	5.76728	0.110
db4	5.47267	0.085	bior1_5	5.44913	0.088	Coif 1	5.26203	0.190
dp5	5.65446	0.098	bior2_2	5.20401	0.081	Coif 2	5.62977	0.180
db6	5.81814	0.170	bior2_4	5.52928	0.094	Coif 3	5.80545	0.099
dp7	5.59607	0.099	bior2_6	5.73171	0.096	Coif 4	5.81171	0.140
dp8	5.55018	0.092	bior2_8	5.74572	0.093	Coif 5	5.7087	0.110
db9	5.62716	0.081	bior3_1	4.04884	0.180	Sym 3	5.44103	0.094
db10	5.82144	0.080	bior3_3	4.04884	0.170	Sym 3	5.37424	0.096
dp11	5.71459	0.098	bior3_5	5.45456	0.090	Sym 4	5.45931	0.097
db12	5.53923	0.190	bior3_7	5.4685	0.098	Sym 5	5.67945	0.093
dp13	5.60735	0.130	bior3_9	5.5201	0.120	Sym 6	5.74788	0.094
db14	5.74366	0.092	bior4_4	5.60627	0.098	Sym 7	5.53356	0.092

**Table 5:** Percentage error for 20-mm and 30-mm lift-off.

Inspection Speed in mm/s	20-mm Lift-off		30-mm Lift-off	
	Experimentally Measured Length in mm	% Error for Length	Experimentally Measured Length in mm	% Error for Length
2.9	31.90	00.55	33.35	3.25
	43.50	06.82	43.50	6.09
	52.20	05.12	52.20	4.40
7.3	33.10	02.47	36.65	13.46
	45.99	12.17	43.80	6.82
	54.97	09.94	54.75	9.50
11	34.10	05.57	38.50	19.19
	46.20	12.68	49.50	20.73
	55.00	10.00	55.00	10.00
13	39.00	20.74	39.00	20.74
	52.00	26.82	53.30	30.00
	58.50	17.00	61.10	22.22

**Table 6:** Percentage error of cavitation corrosion defects for 20-mm lift-off and inspection speed at 2.8 mm/s.

Actual	Experimental	% Error of Length	Actual Depth (mm)	Peak Voltage (V)
40	41.24	3.02	1	2.054
40	40.43	1.07	0.75	1.999
40	41.10	2.75	0.5	1.941

**Table 7:** Percentage error of uniform corrosion defects for 20-mm lift-off and inspection speed at 2.9 mm/s.

Actual	Experimental	% Error for Length	Actual Depth (mm)	Peak Voltage (V)
20	20.417	2.08	2	2.294
20	20.754	3.77	2	2.294
25	24.884	0.4	2	2.294
15	13.790	8.06	2	2.294
